# Studying endocrine disrupting chemicals from molecular targets to mixture model approach: lessons from the thyroid model

**DOI:** 10.1210/clinem/dgag149

**Published:** 2026-04-08

**Authors:** Francesca Coperchini, Alessia Greco, Elena Franchi, Marco Denegri, Mario Rotondi

**Affiliations:** Department of Internal Medicine and Therapeutics, University of Pavia, Pavia 27100, Italy; Department of Internal Medicine and Therapeutics, University of Pavia, Pavia 27100, Italy; Department of Internal Medicine and Therapeutics, University of Pavia, Pavia 27100, Italy; Unit of Endocrinology and Metabolism, Laboratory for Endocrine Disruptors, Istituti Clinici Scientifici Maugeri IRCCS, Pavia 27100, Italy; Department of Internal Medicine and Therapeutics, University of Pavia, Pavia 27100, Italy; Unit of Endocrinology and Metabolism, Laboratory for Endocrine Disruptors, Istituti Clinici Scientifici Maugeri IRCCS, Pavia 27100, Italy

**Keywords:** thyroid, endocrine disruptors, AOP, mixtures

## Abstract

Endocrine-disrupting chemicals (EDCs) pose a significant global health risk by interfering with hormonal balance. The thyroid is particularly suitable for studying endocrine disruption due to its crucial role in development, metabolism, and cognitive function, alongside established molecular targets and regulatory frameworks. This review summarizes current knowledge on thyroid-disrupting chemicals (TDs), focusing on mechanistic pathways acting at both intrathyroidal and extrathyroidal levels. Particular attention is given to molecular initiating events, such as interference with iodide uptake, thyroperoxidase activity, thyroglobulin processing, and thyroid hormone signaling, and to their integration within the adverse outcome pathway (AOP) framework. In addition, the review discusses methodological strategies for assessing thyroid disruption, spanning in silico, in vitro, in vivo, and human epidemiological approaches. Finally, emerging challenges related to real-world exposure to chemical mixtures are addressed, highlighting the need for AOP-informed, mixture-based strategies to improve risk assessment and regulatory decision-making.

The recognition of endocrine-disrupting chemicals (EDCs) began during the era of intense industrialization in the mid-20th century, when large amounts of synthetic compounds were produced and released into the environment under minimal regulation. Since then, scientists began to observe unusual reproductive and developmental changes in wildlife living in contaminated environments (1). These anomalies led researchers to hypothesize that certain environmental chemicals could mimic or interfere with endogenous hormones (2).

Today, endocrine disruptors are widely recognized as a major global health and environmental concern, even though no single international regulatory framework governs their identification and restriction. A growing body of evidence demonstrates that EDC exposure can interfere with multiple endocrine axis, including: the estrogenic and androgenic axis (eg, bisphenol A (BPA), phthalates, diethylstilbestrol (DES)); the glucocorticoid axis (eg, organotins and per- and polyfluoroalkyl substances (PFAS)), the metabolic axis involving insulin sensitivity and adipogenesis (eg, tributyltin, BPA, perfluorooctanoic acid (PFOA)), and the thyroid axis, where several chemicals disrupt hormone synthesis, transport, or signaling (eg, perchlorate, polychlorinated biphenyl substances (PCBs), parabens, PFAS) (3-5). Such interference can lead to health consequences within the exposed individual (intragenerational effects) and across generations (transgenerational effects), as shown in animal models and human epidemiological studies (6-9). Notably, prenatal exposure to chemicals that interfere with thyroid axis has been linked to altered neurodevelopment and impaired cognitive outcomes in offspring (10).

In recent years, international efforts have advanced toward developing harmonized testing strategies and scientifically grounded guidance for EDC assessment. Frameworks such as the Organization for Economic Co-operation and Development (OECD) Guidance Document 150 and the Key Characteristics of Endocrine-Active Chemicals approach (11, 12), now provide structured tools to identify and evaluate endocrine-disrupting potential. Among endocrine targets, the thyroid gland holds a central place due to its crucial role in development, metabolism, growth, and neurocognitive functions. Thyroid hormones (Triiodothyronine, T3, and Thyroxine, T4) are essential for fetal brain development, thermogenesis, and metabolic homeostasis throughout life (4). Consequently, thyroid disruption can have long-lasting and irreversible effects during critical developmental windows. The aim of this review is to summarize the current knowledge on which would be the most exhaustive approach to study thyroid disruptors as well as to provide an overview of how the thyroid disrupting effect may be exerted through different mechanisms acting at different and specific intra and extrathyroid targets. A further aim of the present review will be to overview potential analytical strategies to assess the multifaced effects of endocrine disruptors on thyroid homeostasis.

## Methods

References for this review were identified through searches of PubMed for articles published between January 1971 and June 2025, using keywords related to endocrine disruption and thyroid function, including “endocrine disruptors,” “thyroid disruptors,” “thyroid hormone system,” “adverse outcome pathways,” and “chemical mixtures.” Additional relevant studies and regulatory documents were identified through manual reference screening, Google Scholar, and personal archives. We included experimental, epidemiological, and regulatory-focused articles in English, emphasizing intra and extrathyroidal mechanisms and mixture-based approaches.

## Endocrine disruptors: from myth to reality

### From environmental observation to endocrine science

There is no single universally recognized “first study” that officially formulated the hypothesis that a substance could act as an endocrine disruptor. However, there are some milestones that mark the beginning of scientific awareness of the possibility of endocrine disruption by chemicals. In 1958, the endocrinologist Roy Hertz stated that certain synthetic hormones used in farming could end up in our bodies and mimic endogenous hormones (13).

The publication of “Silent Spring” by Rachel Carson in 1962 marked a pivotal moment in recognizing the dangers of pesticides, leading to increased environmental activism and the establishment of the Environment Protection Agency (EPA). Carson highlighted the harmful effects of dichlorodiphenyltrichloroethane (DDT) on wildlife, linking its accumulation to a decline in fish and bird populations and potential reproductive issues due to reduced estradiol levels. Decades later, it was found that girls exposed to high levels of DDT in utero during the 1960s faced a higher risk of developing breast cancer (14). In 1979, John McLachlan held the first “Oestrogens in the Environment” conference, focusing on the hormonal impact of environmental pollutants. In the 1980s, Colborn et al studied wildlife in the Great Lakes, identifying severe metabolic and reproductive issues in animals exposed to DDT and PCBs. Their findings indicated that despite EPA regulations, harmful xenoestrogens continued to affect ecosystems and animal health (15).

### From skepticism surrounding the phenomenon of “endocrine disruption” to an official recognition

When the first environmental and reproductive anomalies were reported in wildlife between 1970s and 1990s, the notion that synthetic chemicals could interfere with hormonal function was met with profound skepticism for some key reasons. First, early studies revealed nonlinear dose–response relationships that challenged the traditional toxicology principle “the dose makes the poison.” Unlike classical toxicants, endocrine-active compounds can cause significant biological effects at environmentally relevant or even at low doses, especially during critical developmental windows (16). Second, the absence of direct evidence in humans initially hindered acceptance. The earliest findings came from ecological and wildlife observations, such as the feminization of fish exposed to sewage effluents, reproductive failure in birds exposed to DDT, and genital malformations in alligators inhabiting polluted lakes (17). While striking, these studies were often dismissed as species-specific or anecdotal, rather than indicative of a broader biological phenomenon. Third, there was no unifying mechanistic explanation. The implicated substances were highly different from a chemical point of view (including pesticides, industrial PCBs, phthalates, BPA, and organotoxins) and acted through profoundly different pathways. In the absence of a precise molecular target, the idea that this group of compounds could all act via endocrine disruption seemed not plausible (18). By the early 2000s, however, converging lines of evidence from molecular biology, epidemiology, and regulatory science transformed the “myth” into a scientific reality, indeed:

- Molecular evidence identified key receptors, enzymes, and transporters affected by EDCs, such as estrogen and androgen receptors (ER, AR), aromatase, thyroid peroxidase (TPO), and the sodium-iodide symporter (NIS) (19).
- Human cohort studies demonstrated reproducible associations between circulating levels of EDCs and alterations in reproductive, metabolic, and thyroid physiology (20).
- Institutional recognition followed the World Health Organization (WHO) and United Nations Environment Programme (UNEP) officially classified EDCs as crucial environmental contaminants (21). The OECD, EFSA, and the European Commission subsequently established criteria for their identification and regulatory evaluation (22).

EDCs are now a well-defined toxicological category, with established links between molecular mechanisms and adverse outcomes across species and generations. This marks a shift in toxicology, focusing on low-dose, mixture, and developmental effects that impact endocrine homeostasis throughout life.

### The hallmarks of EDCs

When studying EDCs, it is crucial to recognize several distinctive features of their biological behavior that distinguish them from conventional toxicants. First, EDCs frequently exhibit nonmonotonic dose-response relationships, meaning that their effects do not necessarily increase linearly with dose. Instead, both very low and high concentrations can elicit biological effects in opposite directions (15). This biphasic pattern is typical of natural hormones such as estrogens and androgens, where low doses stimulate proliferation while higher doses inhibit it, and reflects the activation of distinct receptor pathways or feedback mechanisms at different concentration ranges (16). A nonmonotonic dose-response curve was defined as following: “*a nonlinear relationship between dose and effect where the slope of the curve changes sign somewhere within the range of doses examined*”, resulting in U-shaped, bell-shaped, or even more complex multiphasic curves. These nonmonotonic effects impair the use of the classical toxicological paradigm, which assumes that the dose-response relationship is monotonic or nonlinear, but without a change in the sign of the slope (16).

**Figure 1:**
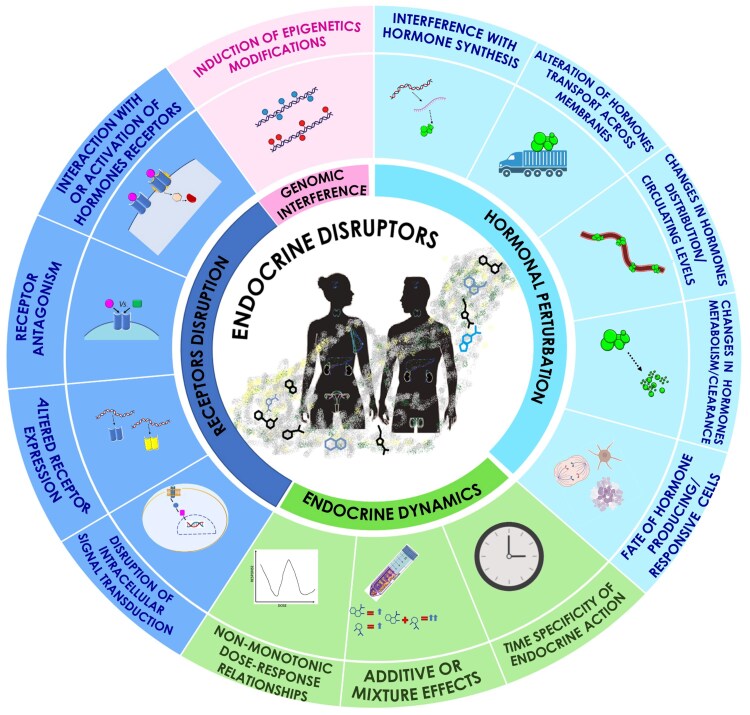
The hallmarks of endocrine disruption. A representation of the hallmarks of endocrine disruption categorized as follows: *Receptor Disruption* encompasses mechanisms related to interference with hormone receptors, including: (1) interaction with or activation of hormone receptors; (2) receptor antagonism; (3) altered receptor expression; and (4) disruption of intracellular signal transduction. *Genomic Interference* refers to mechanisms involving direct or indirect effects on the genome, including: (5) induction of epigenetic modifications. *Hormonal Perturbation* includes processes affecting hormone availability and action, such as: (6) interference with hormone synthesis; (7) alteration of hormone transport across membranes; (8) modification of hormone distribution or circulating levels; (9) changes in hormone metabolism or clearance; and (10) perturbation of the fate of hormone-producing or hormone-responsive cells. A fourth category, “*Endocrine Dynamics”*, captures fundamental properties of endocrine disruption that cut across all mechanisms, including nonmonotonic dose–response relationships, additive or mixture effects, and time specificity of endocrine action. Understanding these mechanisms and the key features of Endocrine Dynamics is crucial for predicting the biological effects of endocrine-disrupting chemical exposure. Figures drawn by using https://smart.servier.com/. Created in BioRender. Rotondi, M. (2026) https://BioRender.com/fihsm2r.

EDCs can mimic or block hormones by binding to multiple receptors, triggering inappropriate signaling (11) even at low concentrations, particularly during critical developmental windows when hormonal regulation is crucial (11). Another critical aspect is the mixture or cocktail effect, whereby simultaneous exposure to multiple EDCs, even at levels below their individual thresholds of effect, can lead to additive or synergistic disruption of endocrine homeostasis (23). Temporal and contextual factors add complexity, as the same hormone or EDC can have opposite effects based on timing, duration, and life stage of exposure. Acute vs chronic administration may lead to different responses, varying between developmental, reproductive, or adult stages (11). These have long been recognized as the main characteristics of EDCs. However, the growing body of literature, together with the availability of more sophisticated experimental models and large cohort studies comparing exposed and unexposed populations, has allowed the identification of additional features that further complete the overall picture of endocrine disruption. As a result, ten key characteristics were recently identified by La Merrill et al as common mechanistic hallmarks of endocrine disruption (11). These key characteristics can be further organized into four overarching categories as showed in Fig. 1.

## Why is the thyroid the perfect model for studying endocrine disruptors?

### The investigation of the adverse outcome pathways

**Figure 2:**
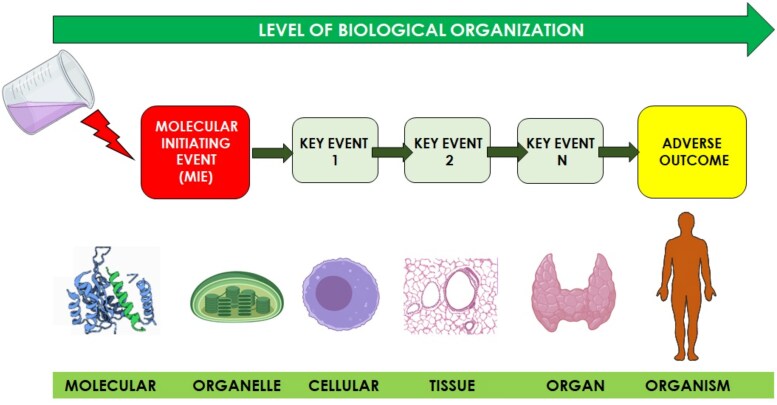
Schematic representation of AOP. **“**Causal maps” illustrate the step-by-step process in which a molecular initial event (MIE) triggered by a chemical substance leads to adverse biological damage relevant to the health of an organism. This occurs through a series of intermediate steps that develop at various levels, including molecular, organelle, cellular, tissue, and organ levels, with key events numbered as 1, 2, 3, and so on. Figures drawn using http://smartserviers.com. Created in BioRender. Rotondi, M. (2026) https://BioRender.com/30ycwos.

The endocrine system is crucial for maintaining internal balance by regulating growth, metabolism, reproduction, and responses to environmental changes through hormones. The thyroid system, in particular, is highly sensitive and offers measurable endpoints at the molecular, cellular, and organism level. Molecular initiating events (MIEs) are well mapped and assayable in vitro, such as inhibition of TPO, blockade of NIS-mediated iodide uptake, displacement of T4 from binding proteins (transthyretin/thyroxine-binding globulin, TTR/TBG), modulation of deiodinases (DIOs) and thyroid hormone receptors, TRα/TRβ, signaling. These mechanisms translate predictably to systemic biomarkers (serum TSH, free T4, free T3) and adverse outcomes (eg, reduced circulating thyroid hormones, altered neurodevelopment). This mechanistic continuity allows the construction of adverse outcome pathways (AOPs) structured frameworks. AOPs are tools from the OECD that link an MIE, such as TPO inhibition or NIS blockade, to a series of biological events, resulting in adverse outcomes for the organism. Essentially, AOPs serve as “causal maps” showing how an initial molecular event leads to significant biological damage affecting health (Fig. 2). A schematic representation of AOP is provided in Fig. 2. AOPs are tools to standardize and make risk assessment predictive. The MIE needs to be measurable, predictable and reproducible. AOPs are particularly valuable in the context of in vitro testing, as they provide a scientifically grounded way to interpret cellular or molecular findings in terms of potential whole-organism toxicity and human health relevance, thus bridging mechanistic assays and regulatory risk assessment. Indeed, assigning the toxic effect of a substance to a specific AOP, represents a crucial step for proceeding toward mechanistic toxicology (24, 25).

### The multi-tier structure of thyroid model

In vivo (animal models), the hypothalamus-pituitary-thyroid (HPT) axis is so conserved that amphibian metamorphosis (a thyroid hormone (TH)-dependent program) serves as a sensitive marker of its functionality (26). At the in vitro level, thyroid cell models provide an excellent system to study endocrine disruptors because key molecular targets of disruption, such as NIS, TPO, thyroglobulin (TG), and TRα/β are well characterized, quantifiable, and functionally conserved between rodents and humans. Established cell lines like FRTL-5 (rat thyrocytes) and Nthy-ori 3-1 (human thyrocytes), and other engineered cell models, retain the ability to respond to TSH and synthesize thyroid-specific proteins, enabling mechanistic evaluation of EDC effects on hormone synthesis, oxidative stress, and gene regulation. These systems allow controlled dose-response experiments and mechanistic dissection that cannot be achieved in vivo, while providing data directly transferrable to human thyroid physiology (27).

Thus, the thyroid model benefits from: (1) clear quantitative clinical readouts (TSH/T4/T3); (2) strong cross-species conservation of mechanism, and (3) regulatory test batteries explicitly built around thyroid homeostasis.

This multi-tier structure (in vitro, amphibian/rodent, human epidemiology) is already codified in OECD GD-150, which prioritize thyroid endpoints for EDC identification (12). Current regulators explicitly embed these endpoints: amphibian metamorphosis (28), repeated-dose studies with thyroid endpoints (29-31), transport/binding and biosynthesis assays, all organized under guidance likewise specifies thyroid disruption assessment (thyroid hormones, NIS/TPO/DIO/TTR, and developmental neuro endpoints) (12). Because thyroid disruption involves multiple mechanisms at several levels (synthesis, transport, receptor binding, metabolism, clearance), a combination of in vitro mechanistic assays, in vivo mechanistic endpoints, and in vivo adverse-outcome endpoints is required (32). Thus, the thyroid is an ideal model for detecting and regulating endocrine disruption due to its extensively validated assays, strong biomarker-outcome links, and regulatory consensus, offering greater sensitivity and clarity than other endocrine glands.

## The thyroid and thyroid disruptors (TD): how to assess thyroid disruption?

The term “endocrine disruptor” was formally coined at the 1991 Wingspread Conference (33). This term was defined in 2002 by the WHO as “*an exogenous substance or mixture that alters function(s) of the endocrine system and consequently causes adverse health effects in an intact organism, or its progeny, or (sub) populations.*” According to the Endocrine Society, an endocrine-disrupting chemical is defined as: “*an exogenous chemical, or mixture of chemicals, that can interfere with any aspect of hormone action.*” (18) Although international agencies have issued validated testing guidelines for thyroid-related endocrine disruption, the term “thyroid disruptor” has not yet been formally and universally defined or recognized in regulatory frameworks. However, it should be acknowledged that this concept remains a matter of scientific debate. Nevertheless, several scientific reviews and consensus papers (including the present one) have adopted and used the term to describe chemicals capable of interfering with one or more processes of thyroid physiology from hormone synthesis, secretion, transport, and metabolism, to receptor activation and cellular responses (11, 18, 34).

### Methodological approaches to assess thyroid disruption

Assessing the thyroid-disrupting potential of chemicals requires an integrated approach that reflects the complexity of the TH axis, encompassing hormone synthesis, release, transport, metabolism, receptor activation, and feedback regulation. Over the past decade, international initiatives have supported the systematic identification and validation of test methods for appropriately evaluating thyroid homeostasis disruption (THSD) (12, 27). A recent comprehensive review by Vergauwen et al (2024) encompassed all available and emerging assays for THSD and classified them according to the OECD Conceptual Framework (Levels 1-5) (12, 27).

#### Level 1, *in silico* approaches

Computational models and quantitative structure–activity relationships (QSARs) predict molecular events in the thyroid pathway, including inhibition of NIS, TPO activity, and TR binding. At least 12 validated models facilitate cost-effective prescreening before further experimental testing. Although powerful, these tools rely on empirical data and are best applied as part of integrated assessment strategies rather than a stand-alone evidence (27).

#### Level 2, in vitro mechanistic assays

The core of thyroid-disruption testing resides at this level. Vergauwen et al catalogued 67 in vitro assays covering multiple biological domains of thyroid homeostasis. These include assays for hormone synthesis (TPO inhibition and NIS activity), transport and binding assays (TTR displacement), receptor-mediated signaling tests (TRα/β transactivation or TSH-receptor cAMP assays), and methods targeting metabolism and clearance, eg, DIOs and hepatic enzyme induction studies. Several of these are now considered “ready for validation.” (27)

#### Levels 3 to 5 in vivo and integrated endpoints

In vivo models provide evidence of systemic and developmental consequences of thyroid disruption. These include amphibian metamorphosis (28), extended fish assays such as XETA or LAGDA (35, 36), and mammalian reproductive or developmental studies that measure circulating T3, T4, and TSH (37, 38).

### Intrathyroidal vs extrathyroidal targets of thyroid disruptors

**Figure 3:**
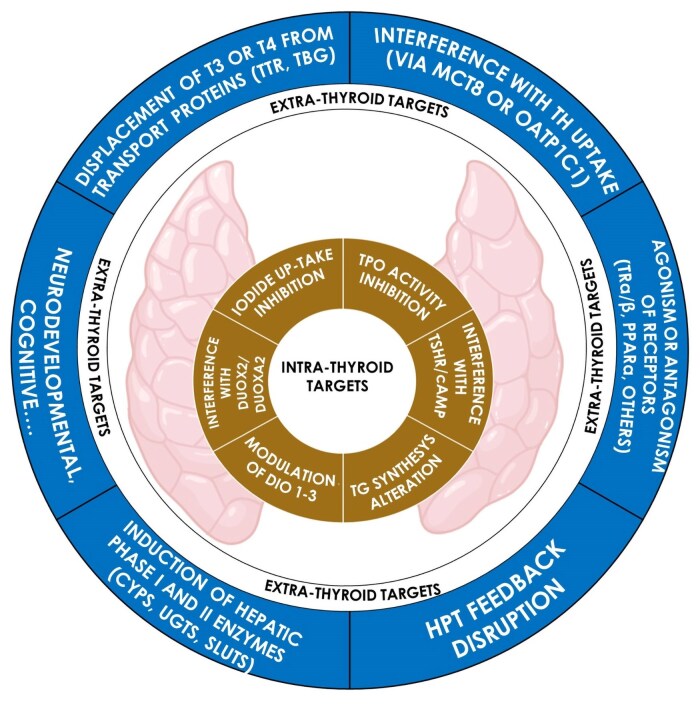
Schematic representation of intra and extrathyroid targets. The internal circle depicts intrathyroidal targets within thyroid follicular cells, including inhibition of iodide uptake, interference with TPO activity and DUOX/DUOXA-mediated H_2_O_2_ generation, alterations in TG synthesis and iodination, modulation of TSH receptor/cAMP signaling, and effects on DIO1-3 activity. The outsider ring illustrates extrathyroidal targets acting outside the thyroid gland, such as displacement of T3 and T4 from plasma transport proteins (eg, TTR, TBG), interference with hormone transporters (eg, MCT8, OATP1C1), induction of hepatic phase I and II enzymes (CYPs, UGTs, SULTs), disruption of HPT axis feedback, receptor agonism or antagonism (TRα/β and related nuclear receptors), and downstream neurodevelopmental effects. Created in BioRender. Rotondi, M. (2026) https://BioRender.com/4mor27d.

Compounds that interfere with thyroid function can act through a wide spectrum of mechanisms, which can be broadly distinguished as intrathyroidal and extrathyroidal targets. This distinction, well established by both literature and international guidance documents (12, 39), reflects whether a compound primarily affects the thyroid gland itself or downstream systemic processes, such as hormone transport/metabolism/receptor-interaction. Investigating these mechanisms is crucial, since interference at these levels constitutes a first measurable step that can trigger downstream biological effects. A summary of intra and extrathyroid targets is provided in Fig. 3.

### Rationale for prioritizing intrathyroidal targets in thyroid disruptor (TD) characterization

When approaching the characterization of a compound suspected to act as a TD, it is generally more appropriate to focus first on ***intrathyroidal targets***. These represent the earliest molecular and cellular events within thyroid follicular cells, the so-called MIE in the AOP framework cited in previous paragraphs (12, 24, 25, 40). An example of thyroid-related AOP is the inhibition of TPO activity. Indeed, this is an MIE from which a series of subsequent key events at cellular, organelles, organ, and organism levels ultimately lead to the final AOP (number 42) (41). It is worth remembering that AOPs are provided and recognized by institutional bodies (24, 42, 43).

Interference with iodide uptake by NIS, TPO, hydrogen peroxide generation (DUOX/DUOXA), TG iodination, TSH receptor signaling, or DIOs activity, all represent measurable perturbations (which are MIEs) that can propagate along the cascade, ultimately leading to thyroid dysfunction. Intrathyroidal endpoints are experimentally accessible, reproducible, and well standardized. In in vitro systems using either human primary thyroid cell cultures and/or cell lines such as FRTL-5 or Nthy-ori 3-1 cells, or by enzymatic assays, intrathyroidal endpoints are useful for hazard identification (44-47). Moreover, alterations detected at this level are strongly correlated with systemic thyroid effects observed in vivo and in human populations (40, 48). Differently, extrathyroidal mechanisms including thyroid hormone transport (TTR, TBG, MCT8, OATP1C1), hepatic metabolism (UGTs, SULTs, CYPs), and receptor-mediated signaling (TRα/β, PPARs, ERs) often require complex or multi-organ models, higher costs, and specialized expertise (49, 50). Thus, starting with intrathyroidal investigations is a pragmatic and scientific approach to establish whether a compound can be classified as a TD and to identify relevant AOP linkages for further mechanistic exploration.

Due to the complexity of these mechanisms, it's unrealistic for one study or laboratory to explore all intra and extrathyroidal targets. Each process needs specific assays, equipment, and biological models, from enzymatic systems to in vivo feedback loops. Thus, most studies examine a subset of targets, focusing on either intrathyroidal or extrathyroidal, to develop a comprehensive mechanistic profile (12, 51, 52).

Assessing thyroid disruption is complex, as compounds can target both intra and extrathyroidal sites. Mechanistic evaluations usually start with early intrathyroidal events. Evidence shows that various environmental compounds, like perchlorate, nitrate, bisphenols, and pesticides, disrupt thyroid homeostasis through different mechanisms (4, 48, 53-55). In addition, it is important to highlight that many chemicals initially identified as TD have later shown mechanistic links to carcinogenesis, through pathways such as chronic TSH stimulation, promotion of oxidative stress, epigenetic alterations, and persistent bioaccumulation (56-59). This highlights the necessity of incorporating thyroid-disruption endpoints into carcinogenicity assessments.

## From single-compound assessment to mixture approach

Modern societies face exposure to numerous commercially registered chemicals. Human-made materials now exceed all living biomass on Earth. Among about 1000 chemicals associated with plastic packaging, over 60 are highly hazardous, with more than 30 identified as EDCs (4). Despite bans on many toxic substances, humans are still exposed to “legacy compounds” that persist due to their durability and high production volumes (55). While exposure to some banned EDCs is gradually decreasing, novel chemicals continue to enter the environment, causing rapidly rising exposure levels (4, 60, 61). As a result, virtually no one can be considered “EDC negative,” making it difficult to establish low-exposure control groups in epidemiological studies. Here follow the main reasons to retain the approach of studying EDC as single compounds, which is nowadays overcome, indeed:

- Human exposure typically involves multiple chemicals at once (61).
- Chemical mixtures can have additive, synergistic, or antagonistic effects, so their impacts cannot always be predicted by evaluating individual chemicals alone (60).
- Traditional toxicology and regulatory policies often rely on single-compound, linear dose-response models, which may not represent real-life exposure to mixtures (62).
- Many endocrine-active chemicals are persistent and bioaccumulative, lasting in the environment or organisms for extended periods. Therefore, historic exposures should be considered (55, 61).
- In terms of thyroid homeostasis, various pathways (synthesis, transport, metabolism, receptor binding) can be impacted, making the term “thyroid disruptor effect” somewhat limited (4, 61, 63).

Mixtures assessment is becoming essential for realistic risk evaluation, rather than focusing on single compounds.

### Current “home-made” strategies to assess the thyroid-disrupting effect of mixtures

Available mixture studies focusing on thyroid disruption are highly heterogeneous, largely because no specific guideline or standardized framework currently exists to define how a good model of thyroid-disrupting mixture should be assembled. Table 1 represents the main strategies used by current studies on TD to compose and investigate the mixture.

**Table 1:** “Home-made” strategies to assess the thyroid-disrupting effect of mixtures

Strategy	Type of compound/target	Description	Reference
***Target-based mixtures (molecular initiating event-driven)***	*SAME FAMILY, SAME TARGET*	Compounds chosen because they hit the same intrathyroidal target (eg, NIS, TPO).	Tonacchera et al (64), Habza-Kowalska et al (65)
***Chemically related mixtures***	*SAME FAMILY, MULTIPLE TARGETS*	Structurally similar compounds grouped together (eg, PBDE congeners in DE-71) vs multiple targets	Kronborg et al (66)
***Outcome-based mixtures***	*DIFFERENT CHEMICALS SAME OUTCOME*	Compounds selected because they cause a shared endocrine phenotype (HPT disruption).	Paiva-Melo et al (67)
***Human bio-monitoring-based mixtures***	*DIFFERENT CHEMICALS SAME OUTCOME*	Compounds grouped because they are present together in environmental matrices (eg, water, soil, dust, or food)	Yue et al (68)

Tonacchera et al (2004) were the first to investigate a true mixture of thyroid-disrupting compounds on an intrathyroidal target (target-based mixture in Table 1), demonstrating that perchlorate, thiocyanate, nitrate, and iodide exert fully additive inhibition of the human NIS. Their investigation established the earliest mathematical approach (perchlorate equivalent concentration) to model mixture effects at the thyroidal MIE level, paving the way for subsequent mixture-based thyroid toxicology (64). Of particular relevance is the study by Habza-Kowalska et al, testing the effects of different phenolic compounds on TPO activity (target-based mixture in Table 1). The authors demonstrated that TPO inhibition cannot be predicted by testing single compounds alone. Indeed, each polyphenol showed a distinct potency and mode of action; binary mixtures produced highly heterogeneous outcomes, including synergistic enhancement, simple additivity, and even strong antagonism (65). These findings showed that the biological effect of a chemical mixture is often unpredictable based on the single components. Kronborg et al used DE-71, a well-characterized commercial mixture of Polybrominated diphenyl ether (PBDE) congeners whose composition reflects the relative proportions found in real human exposure, allowing a toxicologically meaningful assessment of mixture effects (chemically related mixture in Table 1) (66). Their findings demonstrate that this environmentally relevant PBDE mixture markedly suppresses human thyrocyte function reducing: (1) TG secretion; (2) TSH-stimulated cAMP; and (3) the expression of key thyroid-specific genes without inducing overt cytotoxicity. This highlights that mixture-defined exposure, rather than single-congener testing, is essential to capture the true thyroid-disrupting potential of PBDEs (66). Paiva-Melo et al selected their mixture by combining Tributyltin (TBT) and BPS at the same environmentally relevant doses used for the single-compound exposures, allowing a direct comparison and enabling the identification of mixture-specific “emergent” effects that could not be predicted from either chemical alone (67) (outcome-based mixture in Table 1). Their findings show that the TBT + BPS mixture disrupts the HPT axis as assessed by increased T4 levels, reduced T3 levels, marked suppression of TPO, and compensatory up-regulation of DIOs, demonstrating that realistic mixture design reveals biological outcomes fundamentally different from individual exposures. Yue et al conducted a cross-sectional study quantifying 11 urinary EDCs, including phthalate metabolites, BPA analogs, perchlorate, and thiocyanate, to assess their individual and combined effects on THs using multivariable regression models (human bio-monitoring based mixture in Table 1) (68). The mixture was therefore constructed based on measured coexposures rather than theoretical combinations. A significant inverse association with total T4, with bisphenol F emerging as the predominant contributor, was finally found.

### Experimental and computational approaches for mixture assessment

Studies reported in the previous paragraph illustrate various strategies for constructing potential thyroid-disrupting mixtures. These efforts provide guidance on both the assembly of mixtures and their experimental evaluation. Examples of recent research on this topic are reported below.

Hamid et al highlighted the need for optimized experimental strategies for studying ED mixtures. Current approaches (mathematical, factorial, Taguchi, uniform designs) enable simultaneous testing of multiple chemicals and their interactions, reducing bias and better reflecting real-world exposure. Equally crucial is the use of environmentally relevant concentrations, which avoids unrealistic high-dose artifacts and makes toxicity outcomes more translatable to human exposure (69). Mixture toxicology predicts combined effects using computational models, like concentration addition and independent action, addressing interactions from additivity to synergy. Toxicogenomic, proteomic, and metabolomic profiling offer mechanistic insight by identifying pathway-level disruptions caused by mixtures, providing biomarkers that reflect biological impact beyond single endpoints (69).

The predictive model by Caporale et al is notable for integrating biological targets, potencies, and combined low-dose activities of multiple chemicals. This model allows for the quantification of their collective impact on endocrine and thyroid regulation rather than assessing each compound separately. This “convergent disruption” approach reveals that mixtures can exert significant biological effects even when every single component is present at a dose considered safe according to traditional toxicology (62). Such evidence supports the need to shift from single-compound risk assessment toward mixture-based frameworks, particularly when evaluating thyroid-active chemicals whose combined actions may more accurately reflect real-world exposure scenarios (62). Another interesting approach was proposed by Gómez-Olarte et al, who used the ENDOMIX project. This project aims to identify realistic EDC mixtures and evaluate immunotoxicity as a key mechanism. It will validate findings in European mother–child cohorts, use advanced immunotoxicology assays and in vitro models, and conduct multispecies in vivo validation. Additionally, it will involve advanced computational modeling and artificial intelligence to integrate all evidence into a mixture-based risk assessment framework (70).

Current literature on thyroid toxicology mixtures reveals diverse approaches, including target-based combinations, chemically related substances, human coexposure patterns, environmental co-occurrence, and shared endocrine outcomes. However, no single framework has emerged as the gold standard. This methodological heterogeneity underscores the urgent need for an integrated, AOP-informed strategy that aligns real-world exposure with intrathyroidal molecular initiating events, enabling mixture designs that are both biologically meaningful and mechanistically predictive.

## Conclusions and future perspectives

TDs represent an example of how different environmental contaminants (including classic anions, PFAS, bisphenols, pesticides, cosmetic ingredients, and naturally occurring polyphenols) can interfere with multiple steps of TH synthesis and regulation. As highlighted throughout this review, these compounds target key intrathyroidal processes such as iodide uptake, TPO activity, DUOX2-dependent H_2_O_2_ generation, TG synthesis and iodination, TSH receptors-cAMP signaling, and intrathyroidal DIO dynamics. These interactions constitute the earliest MIEs of thyroid-specific AOPs and provide the mechanistic “entry point” that links chemical exposure to systemic and developmental thyroid dysfunction.

Evidence from in vitro systems, animal studies, and human epidemiology consistently shows that no single pathway or single compound fully explains thyroid disruption. Many TDs act pleiotropically, affecting multiple intra and extrathyroidal targets simultaneously and producing cumulative or even synergistic effects on thyroid homeostasis. This complexity is particularly relevant in light of real-world coexposure to chemical mixtures, which is now recognized as the dominant exposure scenario in human populations. Current mixture research is not only limited in number but also highly heterogeneous. Some authors group chemicals by structural classes, others by co-occurrence in human biomonitoring data, and others by environmental matrices or food-chain exposure approaches. At present, no single mixture-construction strategy can be regarded as the main one: each offers a complementary perspective. Establishing a unified framework (integrating environmental occurrence, human exposure levels, and mechanistic relevance) will be essential for advancing thyroid-specific mixture investigation. Importantly, recent methodological contributions have begun to offer guidance not only on how mixtures may be assembled but also on how they can be experimentally studied. Several TDs that were initially classified exclusively as hormonal disruptors (including PFAS, PCBs, certain pesticides, and persistent phenolic compounds) have since been implicated in epigenetic alterations, oxidative stress, tumor microenvironment modulation, chronic TSH stimulation, and proliferative remodeling of thyroid tissue. These mechanisms provide biologically plausible connections between early thyroid dysfunction and long-term carcinogenic outcomes. This has important implications for chemical risk assessment. As regulatory frameworks move toward mechanism-based chemical evaluation, the thyroid will continue to serve as a cornerstone model for endocrine disruption science.

Ultimately, recognizing thyroid disruption not only as an endocrine anomaly but also as a potential contributor to long-term metabolic and neoplastic disease, underscores the urgency of reducing exposure to thyroid-active chemicals and prioritizing preventive public-health strategies.

## Data Availability

Data sharing is not applicable to this article as no datasets were generated or analyzed during the current study.

## Abbreviations

AOP: adverse outcome pathway
AR: androgen receptor
BPA: bisphenol A
BPS: bisphenol S
CYP: cytochrome P450
DDT: dichlorodiphenyltrichloroethane
DES: diethylstilbestrol
DIO: deiodinase
EDC: endocrine-disrupting chemical
EFSA: European Food Safety Authority
EPA: Environment protection Agency
ER: estrogen receptor
HPT: hypothalamus-pituitary-thyroid
MCT8: monocarboxylate transporter 8
MIE: molecular initiating event
NIS: sodium-iodide symporter
OATP1C1: organic anion transporter polypeptide 1C1
OECD: Organization for Economic Co-operation and Development
PBDE: polybrominated diphenyl ether
PCB: polychlorinated biphenyl substance
PPAR: peroxisome proliferator-activated receptor
QSAR: quantitative structure–activity relationship
SULT: hepatic sulfotransferase
T3: triiodothyronine
T4: thyroxine
TBG: thyroxine-binding globulin
TBT: tributyltin
TD: thyroid-disrupting chemical
TG: thyroglobulin
TH: thyroid hormone
THSD: thyroid homeostasis disruption
TPO: thyroid peroxidase
TRα/β: thyroid hormone receptor
TTR: transthyretin
UGT: UDP-glucuronosyltransferase
UNEP: United Nations Environment Programme
WHO: World Health Organization
